# Prognostic impact of lymphocyte to monocyte ratio in patients with myelodysplastic neoplasms/syndromes

**DOI:** 10.1007/s44313-025-00115-0

**Published:** 2025-12-27

**Authors:** Wan-Hsuan Lee, Chien-Chin Lin, Xavier Cheng-Hong Tsai, Chia-Lang Hsu, Chi-Yuan Yao, Feng-Ming Tien, Min-Yen Lo, Yu-Sung Chang, Yuan-Yeh Kuo, Shan-Chi Yu, Ming-Chih Liu, Chang-Tsu Yuan, Mei-Hsuan Tseng, Yen-Ling Peng, Ming Yao, Bor-Sheng Ko, Hwei-Fang Tien, Hsin-An Hou, Wen-Chien Chou

**Affiliations:** 1 Divisions of Hematology, Department of Internal Medicine, National Taiwan University Hospital, Taipei, Taiwan; 2 Department of Internal Medicine, National Taiwan University Hospital, Hsin-Chu Branch, Hsinchu, Taiwan; 3 Graduate Institute of Clinical Medicine, College of Medicine, National Taiwan University, Taipei, Taiwan; 4 Division of General Medicine, Department of Internal Medicine, National Taiwan University Hospital, Taipei, Taiwan; 5 Department of Laboratory Medicine, National Taiwan University Hospital, Taipei, Taiwan; 6 Department of Medical Research, National Taiwan University, Taipei, Taiwan; 7 Department of Internal Medicine, National Taiwan University Hospital Yunlin Branch, Yunlin, Taiwan; 8 Tai-Chen Cell Therapy Center, National Taiwan University Hospital, Taipei, Taiwan; 9 Department of Pathology, National Taiwan University Hospital, Taipei, Taiwan; 10 Department of Pathology, National Taiwan University Hospital Cancer Center Branch, Taipei, Taiwan; 11 Department of Hematological Oncology, National Taiwan University Cancer Center, Taipei, Taiwan; 12 Division of Cell Therapy, Department of Integrated Diagnostics and Therapeutics, National Taiwan University Hospital, Taipei, Taiwan; 13 School of Medicine, National Taiwan University College of Medicine, Taipei, Taiwan; 14 Department of Internal Medicine, Far-Eastern Memorial Hospital, New Taipei, Taiwan

**Keywords:** Myelodysplastic syndromes/neoplasms, Prognosis, Risk stratification, Lymphocyte, Monocyte

## Abstract

**Purpose:**

Myelodysplastic syndromes/neoplasms (MDS) represent a heterogeneous group of clonal hematopoietic disorders with variable prognosis. While several risk models exist, the prognostic role of immune-related biomarkers remains unclear. This study aimed to determine whether the lymphocyte-to-monocyte (L/M) ratio at diagnosis serves as an independent prognostic factor in MDS and to explore its biological correlates.

**Methods:**

A retrospective analysis of 554 patients with primary MDS diagnosed at the National Taiwan University Hospital was conducted. Patients were stratified by an L/M ratio cutoff of 1.5, determined by maximally selected rank statistics. Clinical, cytogenetic, and mutational profiles were assessed. Survival outcomes were analyzed using Kaplan–Meier methods and multivariable Cox regression incorporating IPSS-R, IPSS-M, and WHO-2022/ICC classifications. RNA sequencing was performed on diagnostic bone marrow samples to evaluate transcriptomic differences between groups.

**Results:**

Patients with L/M ratio > 1.5 were younger, had lower platelet counts, more advanced subtypes, and higher frequencies of *STAG2* and *U2AF1* mutations. Elevated L/M ratio was significantly associated with inferior leukemia-free and overall survival, independent of established prognostic models. Adverse prognostic effects were mitigated by allogeneic hematopoietic stem cell transplantation but not by hypomethylating agents. Transcriptomic analysis revealed downregulation of inflammatory pathways (IL-2–STAT5, IL6–JAK–STAT3, interferon responses) and the p53 pathway, along with enrichment of MYC targets in the high L/M group.

**Conclusion:**

An elevated L/M ratio is an independent and readily available biomarker that predicts poor outcomes in MDS. Integration of this parameter into existing risk models may refine prognostication and guide treatment intensity. Transcriptomic findings suggest immune suppression and p53 deregulation underlie its adverse impact, highlighting potential therapeutic avenues.

**Supplementary Information:**

The online version contains supplementary material available at 10.1007/s44313-025-00115-0.

## Introduction

Myelodysplastic syndromes/neoplasms (MDS), a broad category of clonal myeloid disorders, are characterized by dysregulated hematopoiesis, which leads to cytopenia and dysplastic hematopoietic cells. Clinical and genetic heterogeneity, recurrent chromosomal abnormalities, and variable prognostic outcomes are features of MDS [1]. To risk-stratify patients with MDS and direct treatment, several prognostic models have been developed, such as the International Prognostic Scoring System (IPSS) [2], revised IPSS (IPSS-R) [3], molecular IPSS (IPSS-M), World Health Organization (WHO) Classification-based Prognostic Scoring System [4], and MD Anderson Prognostic Scoring System [5]. These models primarily incorporate parameters such as cytopenia severity, cytogenetic abnormalities, bone marrow (BM) blast percentage, gene mutations, and transfusion dependency.

Recent studies have also highlighted the prognostic relevance of peripheral lymphocyte and monocyte counts in solid tumors and hematologic malignancies [6–11]. In MDS, absolute lymphocyte count (ALC) and absolute monocyte count (AMC) at diagnosis are individually associated with patient outcomes [12, 13]. Lymphopenia adversely affects survival in IPSS-M-defined low-risk MDS patients [14], whereas monocytopenia, observed in 29.5% of MDS, correlates with higher blast counts and worse outcomes [12, 13]. These findings suggest that peripheral immune cell profiles may reflect the underlying disease biology and immune dysregulation. Given the interplay between these two values, the lymphocyte-to-monocyte (L/M) ratio presents as a more integrative and robust biomarker; however, studies regarding the prognostic implications of the L/M ratio in patients with MDS are scarce.

## Methods

At the National Taiwan University Hospital (NTUH), data were collected from 554 patients with primary MDS who were diagnosed and treated at NTUH. We retrospectively reviewed a cohort of 554 patients diagnosed with primary MDS based on the WHO-2016 criteria, with subsequent reclassification based on the WHO-2022 and International Consensus Classification (ICC). The survival impact of the L/M ratio was evaluated in the context of the novel classification systems IPSS-R and IPSS-M. To avoid confounding factors, patients with a history of chemotherapy, radiation, or hematologic malignancies were excluded, given the distinct mutational profiles and clinical outcomes of primary and secondary MDS [15]. The TruSight Myeloid Panel and HiSeq platform (Illumina, San Diego, CA, USA) were used to sequence the cryopreserved BM samples and identify mutations in 54 myeloid-related genes [16, 17] (Supplemental Table 1). *TP5*3 copy-neutral loss of heterozygosity and five residual genes (*ETNK1, GNB1, NF1, PPM1D*, and *PRPF8*) identified using the IPSS-M model were not evaluated in this study. By following the manufacturer's instructions, the library was prepared and sequenced to achieve a median read depth of 10,550 x. Variant analysis used databases for somatic mutation annotation and interpretation, including COSMIC v86, dbSNP v151, ClinVar, PolyPhen-2, and SIFT. A variant analysis diagnostic algorithm has been previously described [18]. Polymerase chain reaction (PCR) and fluorescence capillary electrophoresis are required for *FLT*3-ITD analysis due to the limitations of next-generation sequencing (NGS), whereas Sanger sequencing and PCR are required for *KMT2A*-PTD analysis [19, 20]. Cellularity and fibrosis of the BM were evaluated and verified by pathologists using reticulin staining. Cytogenetic analyses were performed according to the International System for Human Cytogenetic Nomenclature in cytogenetic analyses [20, 21].

Following the manufacturer’s instructions, RNA was extracted from diagnostic BM samples (without CD34 + cell isolation), and sequencing libraries were created using the TruSeq Stranded mRNA Library Prep Kit (Illumina). The libraries were subsequently sequenced using a 150-bp paired-end read mode on an Illumina NovaSeq 6000. STAR (v2.7) was used to align the clean reads to the human reference genome GRCh38 after adapter sequencing, and low-quality bases were eliminated from the raw sequencing data using Cutadapt (v3.0) in two-pass mode. Each gene's raw count was determined using GENCODE v28 annotation and was then converted to transcripts per million for additional analysis [22].

The NTUH Research Ethics Committee approved this study (approval number: 20220705RINB). Each participant provided written informed consent in compliance with the Declaration of Helsinki.

### Statistical analysis

Fisher's exact or χ^2^ test for categorical variables and the Mann–Whitney U test for continuous variables were used in the statistical analyses. The time between diagnosis and leukemic transformation, death, or last follow-up was referred to as leukemia-free survival (LFS). The relationship between the date of diagnosis and the last follow-up or death from any cause was known as overall survival (OS). Survival curves were produced using Kaplan–Meier analysis, and the log-rank test was used to determine significance. For both univariable and multivariable analyses, Cox proportional hazards models were used. A time-dependent covariate was thought to be allogeneic hematopoietic stem cell transplantation (HSCT) [23]. Maximally selected rank statistics were applied to determine the optimal cutoff point of the L/M ratio [24, 25]. This approach was a suitable standardized two-sample linear rank statistic to determine the maximum standardized statistics of all potential cutoffs, which offered the best separation of the results into two groups [26, 27]. By selecting replacement samples of the same size from the original dataset, bootstrapping replicated the process of creating samples from an underlying population. The results were tested on individuals excluded from the bootstrap or original samples [28]. All *P* values were two-sided, and at *P* < 0.05, they were deemed statistically significant. IBM SPSS Statistics v23 for Windows was used for all analyses.

## Results

### Clinical characteristics and genetic profiles

The demographic features are presented in Table 1. For the total cohort, the median age was 67.3 years, with a male predominance (63.7%). According to the WHO-2016 classification, half (49.9%) of the patients had MDS with excess blasts (EB), including EB1 (19.9%) and EB2 (30.0%). When classifying patients with ICC, there were 76 (13.7%) and 11 (2.0%) patients with MDS/AML who had myelodysplasia-related gene mutations or myelodysplasia-related cytogenetic abnormalities, respectively (Table 1). A total of 21 (3.8%) and 36 (6.5%) patients met the diagnostic criteria for MDS with mutated *TP53* and MDS/AML with mutated *TP5*3, respectively, based on different blast percentages (Table 1). For the WHO-2022 classification, 83 (15.0%) individuals had hypocellular marrow, 12 (2.2%) had significant BM fibrosis, and were grouped as hypoplastic MDS (MDS-h) or MDS with fibrosis (MDS-f) (Table 1).

**Table 1:** Comparison of clinical characteristics between patients with high (> 1.5) or low (≦1.5) lymphocyte/monocyte ratio

Clinical characters	Total (*n* = 554)	L/M ≤ 1.5 (*n* = 206)	L/M > 1.5 (*n* = 348)	*P* value
Sex				0.273
Female	201 (36.3)	81 (39.3)	120 (34.5)	
Male	353 (63.7)	125 (60.7)	228 (65.5)	
Age*	67.3 (18.4–94.5)	68.6 (19.3–94.5)	66.6 (18.4–94.2)	**0.015**
Laboratory data*				
WBC, × 10^9^/L	3.39 (0.6–32.39)	3.44 (0.6–26.31)	3.36 (0.6–32.39)	0.588
ANC, × 10^9^/L	1.55 (0–23.48)	1.66 (0–15.65)	1.49 (0.01–23.48)	0.238
ALC, × 10^9^/L	1.17 (0.07–10.26)	1.03 (0.07–2.98)	1.28 (0.08–10.26)	** < 0.001**
Monocyote, × 10^9^/L	0.22 (0.01–5.72)	0.36 (0.03–3.54)	0.15 (0.01–5.72)	** < 0.001**
Hb, g/dL	8.1 (2.6–17.1)	8.2 (3.4–14.1)	8.0 (2.6–17.1)	0.197
Platelet, × 10^9^/L	81 (1–721)	148 (7–655)	52 (1–721)	** < 0.001**
BM blast (%)	4.4 (0–19.5)	4.0 (0–19.2)	4.9 (0–19.5)	0.528
PB blast (%)	0 (0–18.6)	0 (0–17.0)	0 (0–18.6)	0.123
IPSS-R				**0.008**
Very low	20 (3.6)	11 (5.3)	9 (2.6)	0.093
Low	152 (27.4)	72 (35.0)	80 (23.0)	**0.003**
Int	141 (25.5)	46 (22.3)	95 (27.3)	0.194
High	116 (20.9)	39 (18.9)	77 (22.1)	0.372
Very high	125 (22.5)	38 (18.5)	87 (25.0)	0.075
IPSS-M				**0.003**
Very low	16 (2.9)	9 (4.4)	7 (2.0)	0.109
Low	119 (21.5)	61 (29.6)	58 (16.7)	** < 0.001**
Moderate low	81 (14.6)	29 (14.1)	52 (14.9)	0.781
Moderate high	83 (15.0)	23 (11.2)	60 (17.2)	0.053
High	92 (16.6)	31 (15.0)	61 (17.5)	0.448
Very high	163 (29.4)	53 (25.7)	110 (31.6)	0.142
2016 WHO classification				** < 0.001**
MDS-5q	5 (0.9)	1 (0.5)	4 (1.1)	0.656
MDS-SLD	79 (14.3)	40 (19.4)	39 (11.2)	**0.008**
MDS-MLD	126 (22.7)	43 (20.9)	83 (23.9)	0.419
MDS-RS-SLD	37 (6.7)	24 (11.7)	13 (3.7)	** < 0.00**1
MDS-RS-MLD	24 (4.3)	12 (5.8)	12 (3.4)	0.184
MDS-EB1	110 (19.9)	38 (18.4)	72 (20.7)	0.522
MDS-EB2	166 (30.0)	48 (23.3)	118 (33.9)	**0.008**
MDS-U	7 (1.3)	0 (0.0)	7 (2.0)	0.050
ICC				**0.002**
MDS	413 (74.5)	159 (77.2)	254 (73.0)	0.273
del(5q)	5 (0.9)	1 (0.5)	4 (1.1)	0.656
mutated *SF3B1*	51 (9.2)	32 (15.5)	19 (5.5)	** < 0.001**
NOS, with SLD	90 (16.2)	41 (19.9)	49 (14.1)	0.073
NOS, with MLD	131 (23.6)	46 (22.3)	85 (24.4)	0.575
EB	115 (20.8)	33 (16.0)	82 (23.6)	**0.034**
mutated *TP53*	21 (3.8)	6 (2.9)	15 (4.3)	0.405
MDS/AML	141 (25.5)	47 (22.8)	94 (27.0)	0.273
MDS-related genes mutations	76 (13.7)	26 (12.6)	50 (14.4)	0.564
MDS-related cytogenetics	11 (2.0)	5 (2.4)	6 (1.7)	0.566
mutated *TP53*	36 (6.5)	13 (6.3)	23 (6.6)	0.890
NOS	18 (3.2)	3 (1.5)	15 (4.3)	0.083
2022 WHO classification				**0.001**
MDS-5q	5 (0.9)	1 (0.5)	4 (1.1)	0.656
MDS-*SF3B1*	67 (12.1)	40 (19.4)	27 (7.8)	** < 0.001**
MDS-h	83 (15.0)	27 (13.1)	56 (16.1)	0.341
MDS-LB	122 (22.0)	52 (25.2)	70 (20.1)	0.159
MDS-IB1	92 (16.6)	30 (14.6)	62 (17.8)	0.588
MDS-IB2	127 (22.9)	35 (17.0)	92 (26.4)	**0.006**
MDS-f	12 (2.2)	5 (2.4)	7 (2.0)	> 0.999
MDS-bi*TP53*	46 (8.3)	16 (7.8)	30 (8.6)	0.725
Treatment				
HMA	147 (26.5)	40 (19.4)	107 (30.7)	**0.004**
Intensive chemotherapy	18 (3.2)	5 (2.4)	13 (3.7)	0.401
Clinical trial	22 (4.0)	7 (3.4)	15 (4.3)	0.595
HSCT	93 (16.8)	31 (15.0)	62 (17.8)	0.400
Supportive care	243 (43.9)	105 (51.0)	138 (39.7)	**0.009**
Other treatment^†^	122 (22.0)	50 (24.3)	72 (20.7)	0.325

*P* values of < 0.05 are statistically significant and are shown in bold

Data are presented as n (%)

^*^Median (range)

^†^Other treatment: include low-dose cytarabine, rabbit-derived anti-thymocyte globulin, cyclosporine, danazol, eltrombopag, erythropoietin-stimulating agents, thalidomide, steroid, venetoclax-based therapy and oral chemotherapy

*Abbreviations*: *ANC* Absolute neutrophil count, *ALC* Absolute lymphocyte count, *BM* Bone marrow, *Hb* Hemoglobin, *HMA* Hypomethylating agent, *HSCT* Allogeneic hematopoietic stem cell transplantation, *ICC* International Consensus Classification, *IPSS-R* Revised international prognosis scoring system, *IPSS-M* Molecular international prognosis scoring system, *L/M* Lymphocyte/monocyte ratio, *MDS-RS* MDS with ring sideroblasts, *MDS-EB* MDS with excess blasts, *MDS-SLD* MDS with single lineage dysplasia, *MDS-MLD* MDS with multilineage dysplasia, *MDS-RS-SLD* MDS with ring sideroblasts and single lineage dysplasia, *MDS-RS-MLD* MDS with ring sideroblasts and multilineage dysplasia, *MDS-U* MDS, unclassifiable, *MDS-5q* MDS with low blasts and isolated 5q deletion, *MDS-SF3B1* MDS with low blasts and *SF3B1* mutation, *MDS-LB and RS MDS* with low blasts and ring sideroblasts, *MDS-LB* MDS with low blasts, *MDS-h* Hypoplastic MDS, *MDS-IB1* MDS with increased blasts-1, *MDS-IB2* MDS with increased blasts-2, *MDS-f* MDS with fibrosis, *MDS-biTP53* MDS with biallelic *TP53* inactivation, *NOS* Not otherwise specified, *PB* Peripheral blood, *WBC* While blood cell count

A total of 68.9% patients had IPSS-R intermediate-(25.5%), high-(20.9%), or very high-risk disease (22.5%), and a total of 61.0% patients had IPSS-M moderately high-(15.0%), high-(16.6%), or very high-risk (29.4%) disease (Table 1). Regarding treatments, 41.7% of patients with EB received hypomethylating agents (HMA, 41.7%) or chemotherapy (5.4%), and 19.4% of patients in the intermediate-, high-, or very-high-risk IPSS-R group underwent allogeneic HSCT.

Overall, 78.2% had at least one gene mutation or cytogenetic abnormality. As shown in Supplemental Table 2, the most common mutation in this cohort was the *ASXL1* mutation (21.3%), followed by *TET2* (15.5%), *SF3B1* (13.9%), *RUNX1* (12.3%), *STAG2* (11.9%), and *TP53* (10.6%). When stratified based on the biological function of the affected genes, mutations in genes involved in epigenetic modifications (45.5%), including DNA methylation-related genes (26.7%) and chromatin-modifying genes (28.7%), were the most common, followed by mutations in the spliceosome complex genes (34.3%).

### Clinical and genetics differences between patients with high or low L/M ratio

**Fig. 1:**
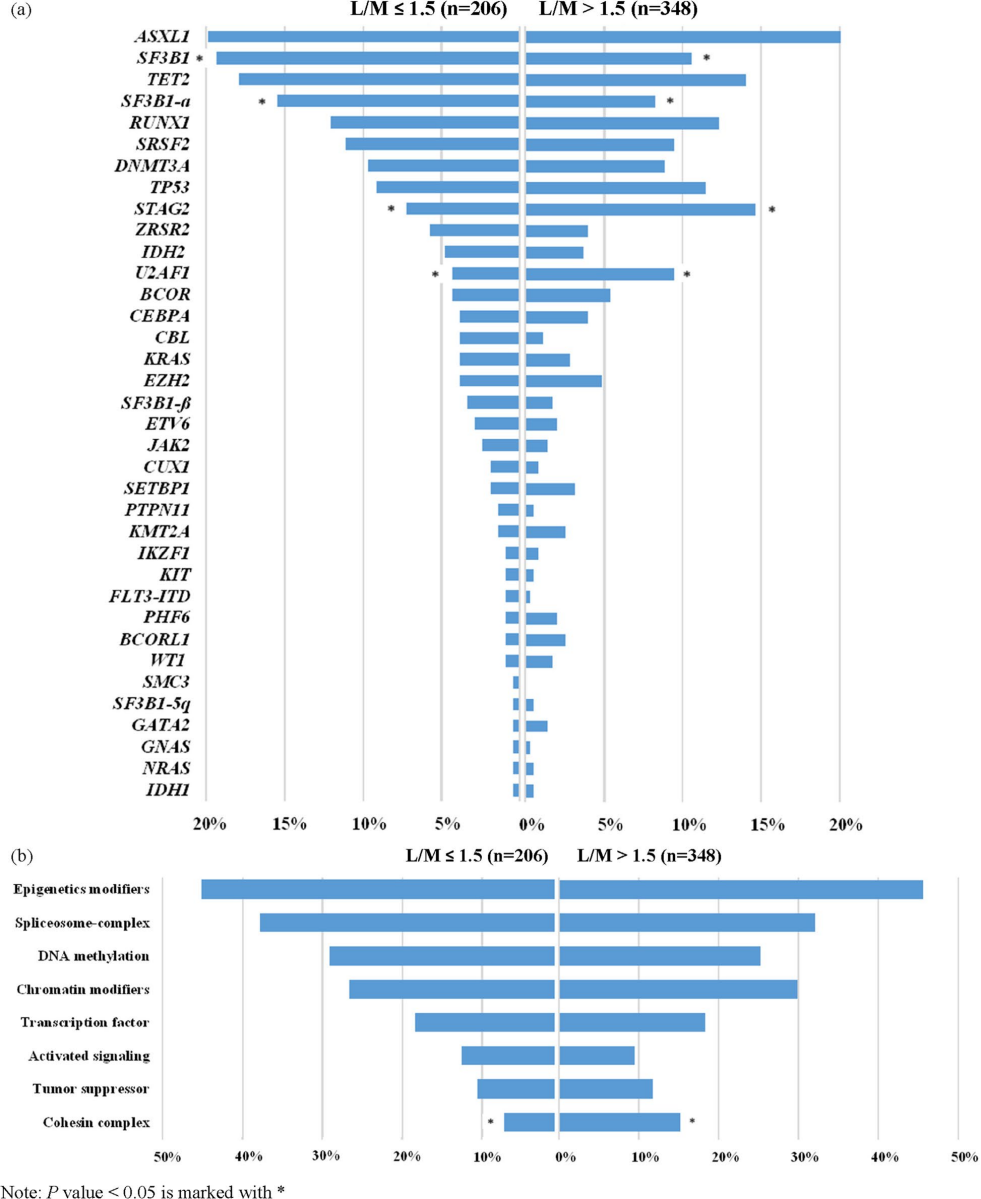
Frequencies of the commonly occurred mutations (**a**) and frequencies of mutations categorized by the functional groups (**b**) in patients with myelodysplastic neoplasms/syndromes

As mentioned above, we used maximally selected rank statistics to determine the optimal L/M ratio cutoff that correlated with the outcomes. Differences in clinical characteristics and genetic profiles between patients with high (> 1.5) or low (≤ 1.5) L/M ratio were explored. Specifically, patients with L/M > 1.5 were significantly younger and had lower platelet counts at diagnosis. They had a higher prevalence of EB2 (WHO-2016), EB (ICC), and IB2 (ICC) subtypes, and a lower prevalence of MDS-SLD, MDS-RS-SLD, and *SF3B1*-mutated subtypes (as defined by both ICC and WHO-2022, Table 1). Taken together, patients with L/M > 1.5 had a lower proportion of low-risk IPSS-R or IPSS-M (Table 1 and Supplemental Fig. 1). Furthermore, those with L/M > 1.5 had more *U2AF1* (9.5% *vs.* 4.4%, *P* = 0.028) and *STAG2* (14.7% *vs.* 7.3%, *P* = 0.009) mutations, while they had less *SF3B1* (10.6% *vs.* 19.3%, *P* = 0.004) mutations compared with those with L/M ≤ 1.5 (Fig. 1 and Supplemental Table 2). When categorized by the genetic functional group, patients with a high L/M ratio had more cohesion complex gene mutations (15.2% *vs.* 7.3%, *P* = 0.007) (Supplemental Table 2 and Fig. 1B).

### Survival impact of L/M ratio

**Fig. 2:**
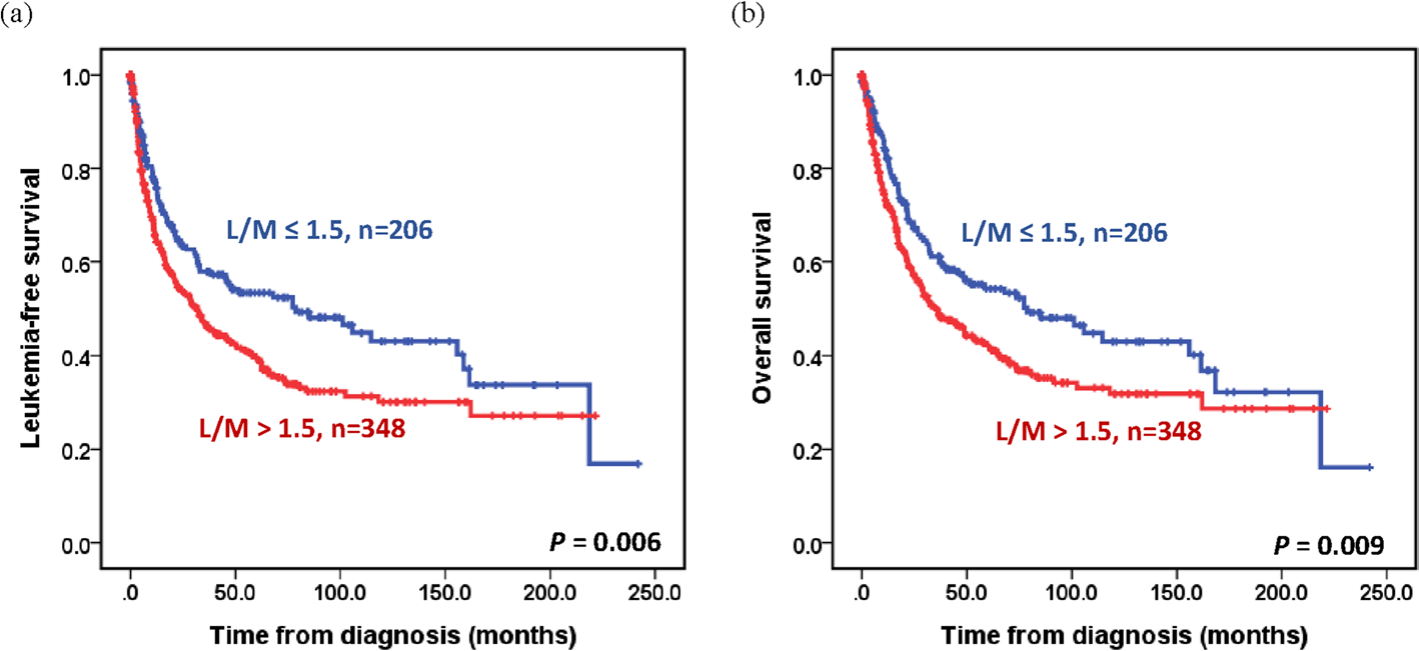
Kaplan–Meier curves for leukemia-free survival and overall survival in patients with myelodysplastic neoplasms/syndromes based on lymphocyte/monocyte (L/M) ratio. **A **Leukemia-free survival, stratified by L/M ratio. **B** Overall survival, stratified by L/M ratio

**Fig. 3:**
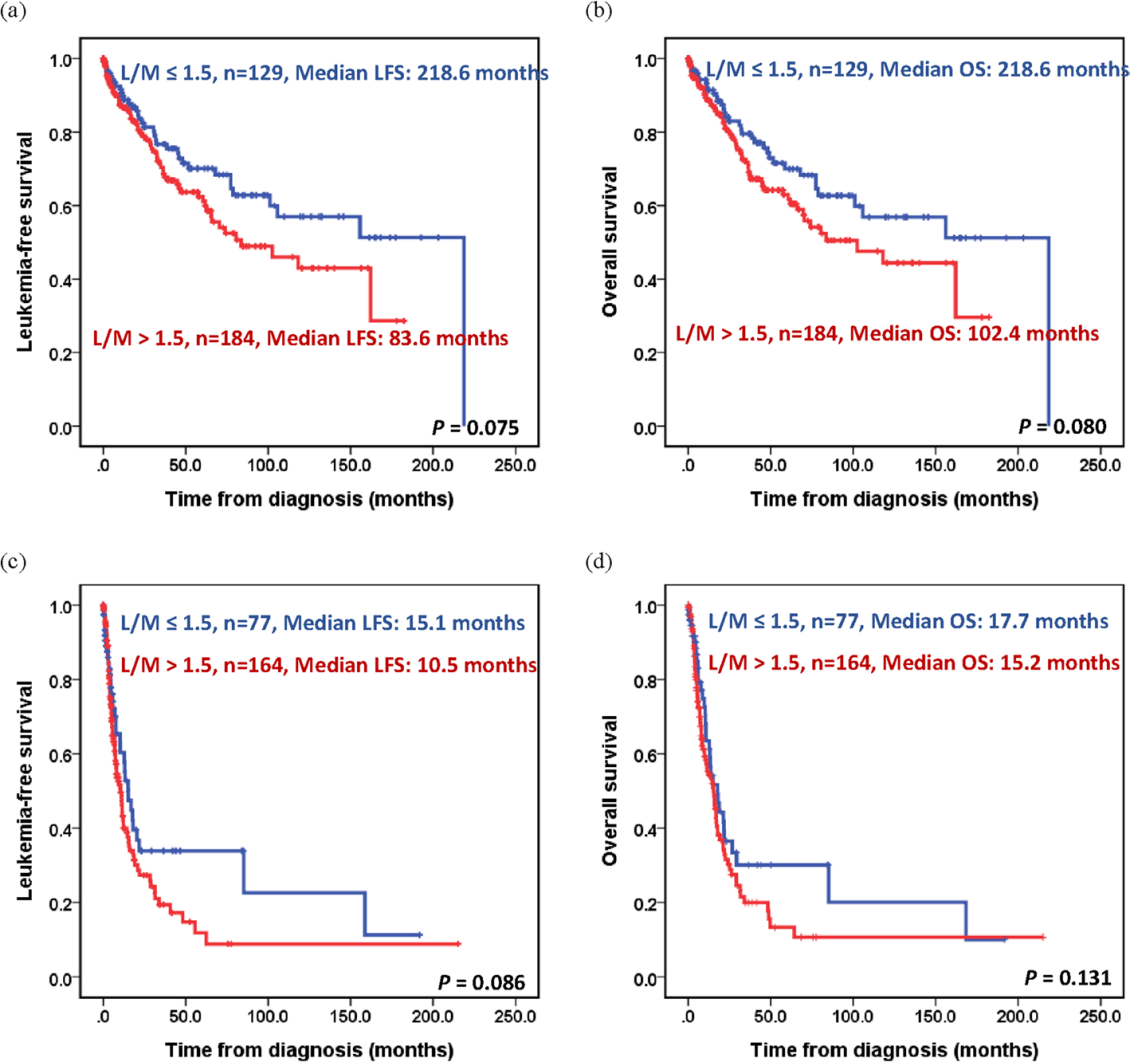
Kaplan–Meier curves censoring at transplantation for leukemia-free survival and overall survival in patients with myelodysplastic neoplasms/syndromes based on lymphocyte/monocyte (L/M) ratio, stratified by revised International Prognostic Scoring System (IPSS-R). **A** Leukemia-free survival, stratified by L/M ratio in patients with very low-, low, or intermediate-risk IPSS-R. **B** Overall survival, stratified by L/M ratio in patients with very low-, low, or intermediate-risk IPSS-R. **C** Leukemia-free survival, stratified by L/M ratio in patients with high, or very high-risk IPSS-R. **D** Overall survival, stratified by L/M ratio in patients with high, or very high-risk IPSS-R

Kaplan–Meier survival analysis showed that patients with L/M ratio > 1.5 had LFS and OS of 31.5 and 34.9 months, respectively, which were significantly shorter than the LFS and OS of those with L/M ratio ≤ 1.5 (78.7 months for LFS and OS, both *P* < 0.05) (Fig. 2). When censoring at the transplantation, individuals with high L/M ratio had a trend of inferior outcomes in both lower (very low, low, or intermediate-risk IPSS-R) or higher (high or very high-risk IPSS-R) risk group (in lower risk group: median LFS: 83.6 *vs.* 218.6 months, *P* = 0.075; median OS: 102.4 *vs.* 218.6 months, *P* = 0.080; in higher risk group: LFS: 10.5 *vs.* 15.1 months, *P* = 0.086; median OS: 15.2 *vs.* 17.7 months, *P* = 0.131) (Fig. 3). According to our previous studies [29, 30], we defined MDS with del5(q), MDS with low blasts (MDS-LB), and MDS-LB and RS as low-risk MDS, whereas MDS with increased blasts and MDS-f were defined as high-risk MDS in the WHO-2022 classification. For the ICC, MDS with del(5q), MDS with mutated *SF3B1*, and MDS, NOS with SLD or MLD were defined as low-risk MDS. The results of Cox regression analyses were internally validated using the bootstrapping method. In univariable analysis, in addition to older age, high ferritin levels, MDS classification based on the ICC or WHO-2022 classification, and risk stratification by the IPSS-R or IPSS-M, L/M > 1.5 was associated with shorter LFS (hazard ratios [HR]: 1.422, *P* = 0.006) and OS (HR: 1.401, *P* = 0.010) (Supplemental Table 3). Furthermore, we validated the thresholds relevant to the prognostic impact of ALC (1.5, or 1.2 × 10^9^/L) and AMC (0.2, or 0.3 × 10^9^/L), which had been reported previously [12, 14, 31, 32] by using our cohort. No differences in survival were observed between the groups. As a continuous variable in the univariable analysis, a higher AMC was associated with shorter LFS and OS (both HR: 1.003, *P* < 0.001) (Supplemental Table 3).

Variables with a *P*-value < 0.1 in univariable Cox regression analysis and allo-HSCT were used as covariates. Two models incorporating ICC or WHO-2022 subtypes were used. For LFS, older age, high ferritin levels, ICC or WHO-2022 subtypes, and higher IPSS-M scores were associated with worse outcomes (all *P* < 0.05). L/M ratio > 1.5 had a trend toward shorter LFS (HR: 1.303, *P* = 0.094; HR: 1.358, *P* = 0.053, Table 2). For OS, older age, L/M > 1.5 (HR: 1.484, *P* = 0.014; HR: 1.548, *P* = 0.006), ICC or WHO-2022 subtypes, and higher IPSS-M were independent poor prognostic factors (Table 2). Additionally, HSCT may confer protective effects (HR: 0.597, *P* = 0.065 for LFS; HR: 0.568,* P* = 0.046 for OS). For patients who did not undergo HSCT, individuals with high L/M ratio had median LFS and OS of 28.8 and 31.3 months, respectively, which were significantly worse than those of patients with a low L/M ratio (median LFS and OS: 78.7 months, both *P* = 0.001, Supplemental Figs. 2A and 2B). At the same time, patients with high L/M ratio receiving HSCT had similar outcomes compared to those with low L/M ratio (median LFS 53.8 and 46.7 months, *P* = 0.755; median OS 73.3 and 73.7 months, *P* = 0.759, Supplemental Figs. 2C and 2D). In patients with very low, low, and intermediate IPSS-R risk, the adverse prognostic impact of a high L/M ratio was mitigated by HSCT (Supplemental Fig. 3). However, HMA treatment could not abrogate the adverse effects of a high L/M ratio (*P* = 0.056 for LFS and *P* = 0.023 for OS).

**Table 2:** Multivariable analysis Cox regression analysis of the impact of different variables on the leukemia-free survival and overall survival of patients with myelodysplastic syndromes/neoplasms

Variable	LFS		OS		LFS		OS	
	**HR (95% CI)**	***P* value**	**HR (95% CI)**	***P* value**	**HR (95% CI)**	***P* value**	**HR (95% CI)**	***P* value**
Age^*^	1.027 (1.016–1.038)	** < 0.001**	1.032 (1.020–1.043)	** < 0.001**	1.027 (1.016–1.038)	** < 0.001**	1.032 (1.021–1.044)	** < 0.001**
**Female**	0.859 (0.627–1.175)	0.341	0.894 (0.649–1.232)	0.494	0.855 (0.624–1.170)	0.327	0.893 (0.648–1.231)	0.491
Ferritin^*^(X 10^**2**^** ng/mL)**	1.001 (1.000–1.001)	**0.022**	1.000 (1.000–1.001)	0.078	1.001 (1.000–1.001)	**0.014**	1.000 (1.000–1.001)	0.065
**L/M > 1.5**	1.303 (0.956–1.775)	0.094	1.484 (1.084–2.031)	**0.014**	1.358 (0.996–1.851)	0.053	1.548 (1.130–2.119)	**0.006**
**ICC**		** < 0.001**		** < 0.001**				
Low-risk MDS^†^	Reference	-	Reference	-				
MDS with EB	1.685 (1.074–2.644)	**0.023**	1.441 (0.905–2.292)	0.123				
MDS/AML^‡^	2.244 (1.344–3.746)	**0.002**	1.840 (1.082–3.131)	**0.024**				
Mutated ***TP53***^§^	4.666 (2.480–8.779)	** < 0.001**	5.743 (2.967–11.118)	** < 0.001**				
**WHO-2022**						** < 0.001**		** < 0.001**
MDS-h, and ***SF3B1***					Reference	-	Reference	-
Low-risk MDS^†^					1.030 (0.649–1.634)	0.901	1.086 (0.683–1.727)	0.726
High-risk MDS^‡^					1.826 (1.082–3.080)	**0.024**	1.592 (0.933–2.718)	0.088
MDS-bi***TP53***					4.588 (2.283–9.220)	** < 0.001**	5.490 (2.664–11.312)	** < 0.001**
**IPSS-M**		** < 0.001**		** < 0.001**		** < 0.001**		** < 0.001**
Very low/low	Reference	-	Reference	-	Reference	-	Reference	-
Moderate low	1.517 (0.854–2.695)	0.155	1.611 (0.908–2.859)	0.103	1.518 (0.852–2.704)	0.157	1.598 (0.898–2.845)	0.111
Moderate high	2.081 (1.224–3.536)	**0.007**	1.952 (1.140–3.344)	**0.015**	2.023 (1.178–3.475)	**0.011**	1.872 (1.080–3.244)	**0.025**
High	2.729 (1.559–4.776)	** < 0.001**	2.898 (1.647–5.099)	** < 0.001**	2.755 (1.561–4.861)	** < 0.001**	2.866 (1.616–5.085)	** < 0.001**
Very high	5.119 (2.857–9.174)	** < 0.001**	4.698 (2.593–8.514)	** < 0.001**	5.536 (3.098–9.896)	** < 0.001**	5.057 (2.804–9.121)	** < 0.001**
**HMA**	0.959 (0.684–1.346)	0.810	0.833 (0.585–1.186)	0.311	1.070 (0.772–1.485)	0.684	0.943 (0.671–1.325)	0.735
**HSCT**	0.597 (0.345–1.032)	0.065	0.778 (0.449–1.349)	0.372	0.568 (0.330–0.976)	**0.040**	0.743 (0.432–1.275)	0.281

*P* values of < 0.05 are statistically significant and are shown in bold

^*^As continuous variables analysis

^†^Low-risk MDS included MDS with del(5q), MDS-*SF3B1*, and MDS, NOS with SLD or MLD

^‡^MDS/AML with MDS-related gene mutations, MDS-related cytogenetic abnormalities, or not otherwise specified

^§^MDS or MDS/AML with mutated *TP53*

*Abbreviations*: *CI* Confidence interval, *EB* Excess blasts, *HR* Hazard ratios, *HMA* Hypomethylating agents, *HSCT* Allogeneic hematopoietic stem cell transplantation, *ICC* International Consensus Classification, *IPSS-M* Molecular International Prognostic Scoring System, *L/M* Lymphocyte/monocyte ratio, *LFS* Leukemia-free survival, *MDS* Myelodysplastic syndromes/neoplasms, *MDS/AML* Myelodysplastic syndromes/acute myeloid leukemia, *OS* Overall survival

Using multivariable analyses, we assessed the prognostic significance of AMC and L/M ratios by combining various variables. Even after controlling for AMC, the L/M ratio remained a valid indicator of poor prognosis for both LFS and OS (Supplemental Table 4). However, without the simultaneous assessment of lymphocyte counts, AMC alone did not demonstrate significant value in independently predicting outcomes (Supplemental Table 5).

### Functional analysis of patients with high or low L/M ratio

**Fig. 4:**
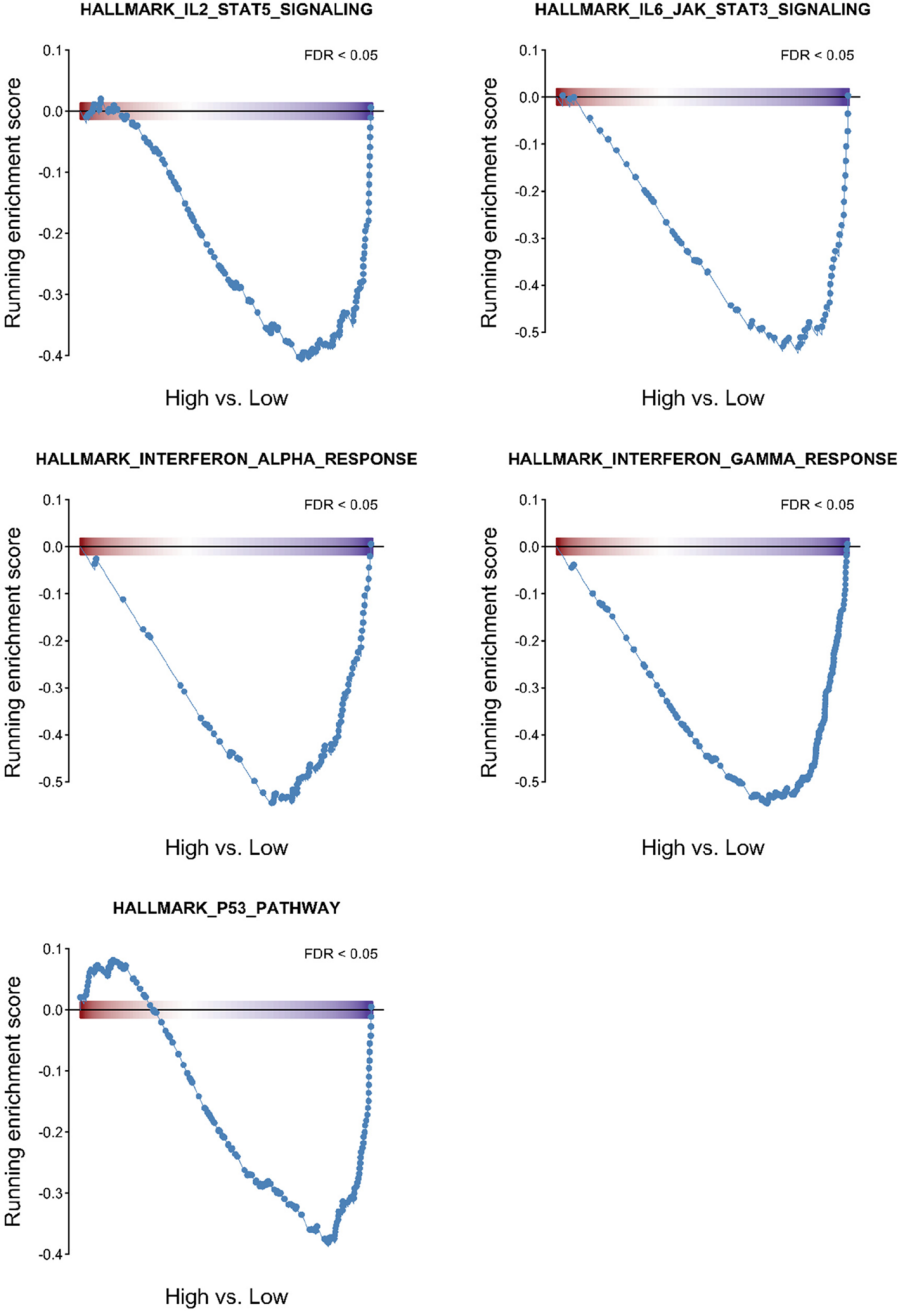
Gene set enrichment analysis highlighted the underexpressed functional pathway in MDS patients with high lymphocyte to monocyte ratio

To clarify the potential biological mechanisms underlying the negative prognostic effect of a higher L/M ratio, we analyzed RNA sequencing data of BM samples from 66 and 44 patients with high and low L/M ratios, respectively. Differential expression analysis between patients with high *vs.* low L/M ratios was performed (Supplemental Fig. 4). The significantly underexpressed functional pathways in patients with a high L/M ratio included IL-2–STAT5, IL6-JAK-STAT3, and interferon-gamma/alpha responses that regulate the inflammatory response (Fig. 4). Similarly, patients with a high L/M ratio showed notable downregulation of the p53 pathway and positive enrichment of MYC target genes (Fig. 4).

## Discussion

This study demonstrated that an elevated L/M ratio of > 1.5 at diagnosis is an independent prognostic factor in patients with MDS, even after adjusting for established risk scoring systems such as IPSS-R and IPSS-M. Furthermore, we assessed the differences in clinical characteristics, genetic profiles, disease subtypes based on the WHO-2016 and WHO-2022 criteria, ICC, and the distribution of risk stratification using the IPSS-R and IPSS-M between patients with high and low L/M ratios. Individuals with a high L/M ratio exhibited higher frequencies of *U2AF1* and *STAG2* mutations but fewer *SF3B1* mutations and were more likely to present with advanced WHO-2022 or ICC subtypes.

In recent years, our knowledge of the pathophysiology underlying MDS has advanced considerably, with disease pathogenesis largely driven by molecular alterations [33–36]. In a more contemporary effort, Bernard et al. proposed the IPSS-M, a prognostic model that integrates clinical parameters, cytogenetic abnormalities, and somatic mutations in 31 genes [37]. The absolute neutrophil count was excluded due to a lack of independent prognostic factors. A six-risk category schema was established, which had a higher prognostic predictive accuracy than the IPSS-R. Previously, we confirmed the prognostic value of the IPSS-M and validated its performance in an Asian cohort [20].

Both the WHO-2022 classification [38] and the novel ICC [39] introduced novel disease entities that incorporated the mutation status of *SF3B1* and *TP53.* Furthermore, the WHO-2022 classification evaluates BM cellularity and fibrosis to define MDS-h and MDS-f. However, the lack of comprehensive genetic sequencing technologies, including NGS or PCR for *KMT2A*-PTD detection, prevents IPSS-M or novel classification systems from being widely used. Because patients with MDS have severe neutropenia and/or neutrophil dysfunction, they are more likely to experience infectious complications. Pollyea et al. discovered that compared to monocytes from healthy control participants, monocytes from patients with MDS had comparatively normal innate immune functions [40]. Furthermore, monocytes from patients with MDS exhibit moderately elevated HLA-DR expression. These findings imply that monocytes help patients with MDS fight infections by compensating for other immune deficiencies [40]. Thus, several studies have documented the negative survival impact of monocytopenia in patients with MDS [12, 13, 31], and it has been linked to negative clinical characteristics, such as greater severity of anemia, neutropenia, and thrombocytopenia [12].

Lymphocyte count is increasingly being recognized as an important prognostic marker in various types of cancer [41–44]. Previous studies have suggested an association between poor prognosis and a lower ALC. Silzle et al. found that lymphopenia < 1.2 × 10^9^/l at diagnosis was associated with inferior outcomes in patients with MDS with low-risk IPSS-R [32]. In the very low- and low-risk IPSS-M groups, ALC < 1.5 × 10^9^/l was correlated with other severe cytopenias, fewer *SF3B1* mutations, and shorter OS [14]. In WHO-2016 classification-defined MDS with ring sideroblasts, Mangaonkar et al. confirmed the negative prognostic predictive value of lymphopenia [45]. Additionally, we validated the prognostic impacts of AMC and ALC, which revealed that AMC did not have a survival effect as a dichotomous variable. In contrast, as a continuous variable, a higher AMC conferred poor outcomes in the univariable analysis. When using a cutoff value of 1.2 × 10^9^/L or 1.5 × 10^9^/L, no survival differences were found. The L/M ratio serves as a significant prognostic biomarker in various cancers, reflecting the interplay between the immune system and tumor biology. In the multivariable analysis, when considering the survival impact of lymphocytes and monocytes, significance was retained for the L/M ratio but not for AMC. This indicated that host immunity may be considered as a factor to incorporate into current risk stratification models, and lymphocytes and monocytes should be evaluated concomitantly. In summary, the current study adds to this knowledge by showing that the L/M ratio is a more accurate prognostic marker than AMC or ALC alone.

In the past few years, various combinations of inflammatory parameters, including the ratios of neutrophils to lymphocytes, platelets to lymphocytes, and lymphocytes to monocytes, have been used to predict the prognosis of patients with hematological and oncological cancers [46–48]. One of the new inflammatory indices, the hemoglobin, albumin, lymphocyte, and platelet (HALP) score, can predict the outcomes of lymphoma, kidney, and lung cancers [49–51]. Gursoy et al. showed that a high HALP score was linked to adverse clinicopathological features in patients with MDS [43].

In our study, we revealed the prognostic implications of the L/M ratio in the context of two novel classification systems (WHO-2022 or ICC). Patients with an L/M ratio > 1.5 had more severe thrombocytopenia and a higher risk of mutational profile with more *U2AF1, STAG2*, but fewer *SF3B1* mutations. Furthermore, we found that HSCT could improve the survival of patients with a high L/M ratio. Thus, these routine laboratory tests may be widely applied and may help identify patients who may benefit from more aggressive therapy.

To further clarify the mechanism underlying the adverse prognostic impact of a high L/M ratio in patients with MDS, we performed transcriptomic analysis and depicted differential gene expression. Gene set enrichment analysis revealed the downregulation of multiple immune and inflammatory signaling pathways, including interferon-alpha and gamma responses, IL6-JAK-STAT3, IL-2–STAT5, and the p53 pathway. These findings suggest a state of immunosuppression or immune evasion in patients with high L/M ratios, which may contribute to disease progression. The underexpression of the interferon signaling axis, particularly type I interferons, may impair antigen presentation and immune surveillance, thereby promoting leukemic clonal expansion [52]. *STAT5* is essential for the development and functional maturation of multiple hematopoietic lineages, including B cells, T cells, natural killer cells, and erythroid progenitors. Loss-of-function *STAT5* mutations are linked to impaired B cell adaptive immunity, immunosuppressive effects, and serious infections, which may contribute to poor outcomes [53]. Abnormal Stat3/5 signaling biosignature in patients with MDS has been reported to predict treatment response and outcomes [54]. The p53 pathway, a key tumor suppressor network, plays a critical role in genomic stability. Loss or dysfunction of p53 leads to enhanced self-renewal of leukemia-initiating cells [55] and evasion of cancer surveillance [56]. Downregulation of the p53 pathway in patients with high L/M ratios may further imply impaired apoptotic regulation and increased genomic instability. MYC expression increased in patients with a high L/M ratio. Higher MYC expression is associated with the blockade of myeloid cell differentiation [57], cooperation with other oncogenes [58], and cancer metabolism [59], which may result in accelerated disease progression and reduced survival [60]. Together, these results provide molecular insights into the adverse prognosis of patients with MDS with a high L/M ratio and highlight the potential utility of immunomodulatory or p53-targeted strategies in this population. The study's retrospective design, inability to assess *TP53* copy-neutral loss of heterozygosity, the requirement of five residual genes by the IPSS-M, and treatment regimen variability are some of its limitations. Although the survival effect of the L/M ratio was internally validated using the bootstrapping method, external validation is warranted to confirm our results. The cutoff of 1.5 in current study may represent an internally optimized threshold rather than a universal reference value. In addition, owing to the retrospective nature of our cohort and incomplete time-to-event data in certain censored cases, reliable estimation of the C-index was not feasible in this study. The underlying pathophysiology of a high L/M ratio leading to poor prognosis requires further exploration. Future studies incorporating comprehensive immune profiling and prospectively standardized follow-up are warranted to quantitatively assess the incremental value of the L/M ratio in risk stratification and to better define its clinical utility—for example, identifying high-risk patients within the same IPSS-M category or optimizing the timing of allogeneic HSCT. However, our study provides a simple and highly applicable method for further identification of patients with MDS who are at a higher risk of progression. These findings have significant clinical implications in resource-limited settings. To find a cure, patients with high L/M ratios may be eligible for more intensive therapies.

In conclusion, this study presents compelling evidence for the prognostic value of the L/M ratio in MDS, advocating its integration into clinical practice. Transcriptomic profiling suggests that altered immune signaling and deregulated oncogenic pathways may have clinical implications. The L/M ratio is a simple and easy-to-use laboratory test that helps differentiate patients with different survival rates, which may potentially improve patient outcomes. Further validation in larger prospective cohorts is essential to confirm their roles in guiding treatment decisions.

## Supplementary Information

Supplementary Material 1

## Data Availability

The datasets generated and analyzed in this study are available from the corresponding author upon reasonable request.
