# Supplementary material for: Prognostic impact of lymphocyte to monocyte ratio in patients with myelodysplastic neoplasms/syndromes
Source: Blood Res. 2025 Dec 27;61(1):6. doi: 10.1007/s44313-025-00115-0 (PMC12852561; doi:10.1007/s44313-025-00115-0)

**Supplemental Table 1. List of 54 myeloid neoplasm-relevant genes studied in targeted NGS sequencing**

| **Gene name** | **Target region (exon)** | **Gene name** | **Target region (exon)** |
| --- | --- | --- | --- |
| ***ABL*** | 4-6 | ***JAK3*** | 13 |
| ***ASXL1*** | 12 | ***KDM6A*** | full |
| ***ATRX*** | 8-10, 17-31 | ***KIT*** | 2, 8-11, 13, 17 |
| ***BCOR*** | full | ***KRAS*** | 2, 3 |
| ***BCORL1*** | full | ***MLL*** | 5-8 |
| ***BRAF*** | 15 | ***MPL*** | 10 |
| ***CALR*** | 9 | ***MYD88*** | 3-5 |
| ***CBL*** | 8, 9 | ***NOTCH1*** | 26-28, 34 |
| ***CBLB*** | 9, 10 | ***NPM1*** | 12 |
| ***CBLC*** | 9, 10 | ***NRAS*** | 2, 3 |
| ***CDKN2A*** | full | ***PDGFRA*** | 12, 14, 18 |
| ***CEBPA*** | full | ***PHF6*** | Full |
| ***CSF3R*** | 14-17 | ***PTEN*** | 5, 7 |
| ***CUX1*** | full | ***PTPN11*** | 3, 13 |
| ***DNMT3A*** | full | ***RAD21*** | Full |
| ***ETV6*** | full | ***RUNX1*** | Full |
| ***EZH2*** | full | ***SETBP1*** | 4 (partial) |
| ***FBXW7*** | 9-11 | ***SF3B1*** | 13-16 |
| ***FLT3*** | 14, 15, 20 | ***SMC1A*** | 2, 11, 16, 17 |
| ***GATA1*** | 2 | ***SMC3*** | 10, 13, 19, 23, 25, 28 |
| ***GATA2*** | 2-6 | ***SRSF2*** | 1 |
| ***GNAS*** | 8, 9 | ***STAG2*** | full |
| ***HRAS*** | 2, 3 | ***TET2*** | 3-11 |
| ***IDH1*** | 4 | ***TP53*** | 2-11 |
| ***IDH2*** | 4 | ***U2AF1*** | 2, 6 |
| ***IKZF1*** | full | ***WT1*** | 7, 9 |
| ***JAK2*** | 12, 14 | ***ZRSR2*** | full |

**Supplemental Table 2. Comparison of genetic alterations between patients with high (>1.5) or low (≤1.5) lymphocyte/monocyte ratio**

| Clinical characters | Total  (n=554) | L/M ≤ 1.5  (n=206) | L/M > 1.5  (n=348) | *P* value |
| --- | --- | --- | --- | --- |
| Epigenetics modifiers | 252 (45.5) | 93 (45.1) | 159 (45.7) | 0.930 |
| DNA methylation | 148 (26.7) | 60 (29.1) | 88 (25.3) | 0.323 |
| *DNMT3A* | 51 (9.2) | 20 (9.7) | 31 (8.9) | 0.753 |
| *TET2* | 86 (15.5) | 37 (18.0) | 49 (14.1) | 0.223 |
| *IDH1* | 3 (0.5) | 1 (0.5) | 2 (0.6) | >0.999 |
| *IDH2* | 23 (4.2) | 10 (4.9) | 13 (3.7) | 0.523 |
| *WT1* | 8 (1.4) | 2 (1.0) | 6 (1.7) | 0.716 |
| Chromatin modifiers | 159 (28.7) | 55 (26.7) | 104 (29.9) | 0.439 |
| *ASXL1* | 118 (21.3) | 41 (19.9) | 77 (22.1) | 0.537 |
| *EZH2* | 25 (4.5) | 8 (3.9) | 17 (4.9) | 0.578 |
| *KMT2A* | 12 (2.2) | 3 (1.5) | 9 (2.6) | 0.549 |
| *SETBP1* | 15 (2.7) | 4 (1.9) | 11 (3.2) | 0.589 |
| *BCOR* | 28 (5.1) | 9 (4.4) | 19 (5.5) | 0.566 |
| *BCORL1* | 11 (2.0) | 2 (1.0) | 9 (2.6) | 0.225 |
| *PHF6* | 9 (1.6) | 2 (1.0) | 7 (2.0) | 0.495 |
| Activated signaling | 59 (10.6) | 26 (12.6) | 33 (9.5) | 0.257 |
| *FLT3-*ITD | 3 (0.5) | 2 (1.0) | 1 (0.3) | 0.559 |
| *FLT3-*TKD | 2 (0.4) | 0 (0.0) | 2 (0.6) | 0.532 |
| *KIT* | 4 (0.7) | 2 (1.0) | 2 (0.6) | 0.631 |
| *KRAS* | 3 (0.5) | 1 (0.5) | 2 (0.6) | >0.999 |
| *NRAS* | 18 (3.2) | 8 (3.9) | 10 (2.9) | 0.621 |
| *PTPN11* | 5 (0.9) | 3 (1.5) | 2 (0.6) | 0.366 |
| *JAK2* | 10 (1.8) | 5 (2.4) | 5 (1.4) | 0.512 |
| *CBL* | 12 (2.2) | 8 (3.9) | 4 (1.2) | **0.065** |
| *GNAS* | 2 (0.4) | 1 (0.5) | 1 (0.3) | >0.999 |
| Transcription factor | 102 (18.4) | 38 (18.4) | 64 (18.4) | >0.999 |
| *CEBPA* | 22 (4.0) | 8 (3.9) | 14 (4.0) | >0.999 |
| *RUNX1* | 68 (12.3) | 25 (12.1) | 43 (12.4) | >0.999 |
| *GATA2* | 6 (1.1) | 1 (0.5) | 5 (1.4) | 0.420 |
| *ETV6* | 13 (2.4) | 6 (2.9) | 7 (2.0) | 0.566 |
| *IKZF1* | 5 (0.9) | 2 (1.0) | 3 (0.9) | >0.999 |
| Spliceosome-complex | 190 (34.3) | 78 (37.9) | 112 (32.2) | 0.195 |
| *U2AF1* | 42 (7.6) | 9 (4.4) | 33 (9.5) | **0.028** |
| *SRSF2* | 56 (10.1) | 23 (11.2) | 33 (9.5) | 0.526 |
| *ZRSR2* | 26 (4.7) | 12 (5.8) | 14 (4.0) | 0.332 |
| *SF3B1* | 77 (13.9) | 40 (19.4) | 37 (10.6) | **0.004** |
| *SF3B1^5q^* | 3 (0.5) | 1 (0.5) | 2 (0.6) | >0.999 |
| *SF3B1^α^* | 61 (11.0) | 32 (15.5) | 29 (8.3) | **0.009** |
| *SF3B1^β^* | 13 (2.3) | 7 (3.4) | 6 (1.7) | 0.208 |
| Cohesin complex | 68 (12.3) | 15 (7.3) | 53 (15.2) | **0.007** |
| *RAD21* | 2 (0.4) | 0 (0.0) | 2 (0.6) | 0.532 |
| *SMC1A* | 1 (0.2) | 0 (0.0) | 1 (0.3) | >0.999 |
| *SMC3* | 1 (0.2) | 1 (0.5) | 0 (0.0) | 0.372 |
| *STAG2* | 66 (11.9) | 15 (7.3) | 51 (14.7) | **0.009** |
| Tumor suppressor | 63 (11.4) | 22 (10.7) | 41 (11.8) | 0.693 |
| *TP53* | 59 (10.6) | 19 (9.2) | 40 (11.5) | 0.402 |
| *CUX1* | 7 (1.3) | 4 (1.9) | 3 (0.9) | 0.433 |
| Median number of mutations | 1 (0-8) | 1 (0-7) | 1 (0-8) | 0.800 |

*P* values of <0.05 are statistically significant.

Data are presented as n (%).

Abbreviations: L/M, lymphocyte/monocyte ratio

Note: Large insertion in *FLT3*-ITD and high GC content in *CEBPA* limited the detection and quantification by NGS.

**Supplemental Table 3. Univariable Cox regression analysis for leukemia-free survival and overall survival**

| Variables | LFS | | OS | |
| --- | --- | --- | --- | --- |
|  | **HR (95% CI)** | ***P* value** | **HR (95% CI)** | ***P* value** |
| Age^*^ | 1.022 (1.014-1.030) | **<0.001** | 1.025 (1.017-1.033) | **<0.001** |
| Female | 0.698 (0.542-0.899) | **0.005** | 0.677 (0.522-0.878) | **0.003** |
| Ferritin^*^ (X 10^2^ ng/mL) | 1.001 (1.000-1.001) | **0.001** | 1.001 (1.000-1.001) | **0.012** |
| L/M > 1.5 | 1.422 (1.107-1.827) | **0.006** | 1.401 (1.085-1.810) | **0.010** |
| AMC (X 10^7^ /L)^*^ | 1.003 (1.002-1.005) | **<0.001** | 1.003 (1.001-1.005) | **<0.001** |
| WHO-2022 classification |  | **<0.001** |  | **<0.001** |
| MDS-h, and *SF3B1* | Reference | - | Reference | - |
| Low-risk MDS^†^ | 1.314 (0.872-1.982) | **0.192** | 1.370 (0.906-2.072) | 0.135 |
| High-risk MDS^‡^ | 4.314 (3.051-6.099) | **<0.001** | 3.812 (2.679-5.424) | **<0.001** |
| MDS-bi*TP53* | 15.075 (9.348-24.311) | **<0.001** | 17.686 (10.845-28.841) | **<0.001** |
| ICC |  |  |  |  |
| Low-risk MDS^§^ | Reference | - | Reference | - |
| MDS with EB | 2.952 (2.162-4.031) | **<0.001** | 2.613 (1.900-3.593) | **<0.001** |
| MDS/AML^¶^ | 4.780 (3.482-6.562) | **<0.001** | 3.924 (2.829-5.442) | **<0.001** |
| Mutated *TP53^#^* | 13.909 (9.332-20.730) | **<0.001** | 16.642 (11.034-25.098) | **<0.001** |
| IPSS-M |  | **<0.001** |  | **<0.001** |
| Very low/low | Reference | - | Reference | - |
| Moderate low | 1.695 (1.022-2.810) | **0.041** | 1.710 (1.031-2.835) | **0.038** |
| Moderate high | 2.608 (1.651-4.118) | **<0.001** | 2.281 (1.428-3.642) | **0.001** |
| High | 4.101 (2.667-6.308) | **<0.001** | 3.915 (2.538-6.040) | **<0.001** |
| Very high | 10.830 (7.290-16.090) | **<0.001** | 9.411 (6.321-14.010) | **<0.001** |
| IPSS-R |  | **<0.001** |  | **<0.001** |
| Very low/low | Reference | - | Reference | - |
| Intermediate | 1.709 (1.181-2.472) | **0.004** | 1.568 (1.076-2.285) | **0.019** |
| High | 3.664 (2.591-5.181) | **<0.001** | 3.234 (2.275-4.595) | **<0.001** |
| Very high | 6.662 (4.725-9.393) | **<0.001** | 6.322 (4.470-8.941) | **<0.001** |
| HSCT | 0.862 (0.586-1.268) | 0.451 | 0.814 (0.551-1.203) | 0.301 |
| HMA | 1.978 (1.546-2.526) | **<0.001** | 1.745 (1.356-2.246) | **<0.001** |

Note: Only 16 (2.9%) and 20 (3.6%) patients were categorized as very low-risk IPSS-M or IPSS-R respectively and there was no inter-group difference between IPSS-M or IPSS-R very low and low risk subgroups in both OS and LFS; accordingly, we put IPSS-M amd IPSS-R very low and low groups together.

*P* values of <0.05 are statistically significant.

*As continuous variables analysis.

^†^Low-risk MDS included MDS with del(5q), MDS-LB, MDS-LB and RS

^‡^High-risk MDS included MDS-IB1, MDS-IB2, MDS-f

^§^Low-risk MDS includes MDS with del(5q), MDS-*SF3B1*, and MDS, NOS with SLD or MLD.

^¶^MDS/AML includes MDS/AML with MDS-related gene mutations, MDS/AML with MDS-related cytogenetic abnormalities, or MDS/AML, not otherwise specified

*^#^*MDS or MDS/AML with mutated *TP53*

Abbreviations: AMC, absolute monocyte counts; CI, confidence interval; EB, excess blasts; HR, hazard ratio; HMA, hypomethylating agents; HSCT, allogeneic hematopoietic stem cell transplantation; h, hypoplastic; ICC, International Consensus Classification; IPSS-M, Molecular International Prognostic Scoring System; IPSS-R, Revised International Prognostic Scoring System; L/M, lymphocyte/monocyte ratio; LFS, leukemia-free survival; MDS, myelodysplastic syndrome/neoplasms; MDS/AML, myelodysplastic syndromes/acute myeloid leukemia; OS, overall survival.

**Supplemental Table 4.** **Multivariable analysis Cox regression analysis of the impact of different variables on the leukemia-free survival and overall survival of patients with myelodysplastic syndromes/neoplasms**

| Variable | LFS | | OS | | LFS | | OS | |
| --- | --- | --- | --- | --- | --- | --- | --- | --- |
|  | **HR (95% CI)** | ***P* value** | **HR (95% CI)** | ***P* value** | **HR (95% CI)** | ***P* value** | **HR (95% CI)** | ***P* value** |
| Age^*^ | 1.027 (1.016-1.038) | **<0.001** | 1.036 (1.024-1.048) | **<0.001** | 1.027 (1.016-1.038) | **<0.001** | 1.036 (1.024-1.048) | **<0.001** |
| Female | 1.150 (0.833-1.588) | 0.396 | 1.057 (0.763-1.463) | 0.739 | 1.155 (0.836-1.597) | 0.382 | 1.059 (0.763-1.468) | 0.733 |
| Ferritin^*^(X 10^2^ ng/mL) | 1.000 (1.000-1.001) | **0.037** | 1.000 (1.000-1.001) | 0.117 | 1.001 (1.000-1.001) | **0.021** | 1.000 (1.000-1.001) | **0.086** |
| L/M > 1.5 | 1.404 (1.020-1.931) | **0.037** | 1.558 (1.128-2.151) | **0.007** | 1.440 (1.047-1.982) | **0.025** | 1.600 (1.158-2.211) | **0.004** |
| AMC | 1.002 (1.000-1.005) | **0.060** | 1.002 (0.999-1.004) | 0.214 | 1.002 (0.999-1.004) | 0.123 | 1.001 (0.999-1.004) | 0.387 |
| ICC |  | **<0.001** |  | **<0.001** |  |  |  |  |
| Low-risk MDS^†^ | Reference | - | Reference | - |  |  |  |  |
| MDS with EB | 1.616 (1.017-2.567) | **<0.001** | 1.310 (0.816-2.104) | 0.364 |  |  |  |  |
| MDS/AML^‡^ | 2.454 (1.467-4.101) | **<0.001** | 1.920 (1.134-3.252) | **0.015** |  |  |  |  |
| Mutated *TP53*^§^ | 4.712 (2.455-9.043) | **0.011** | 5.576 (2.838-10.953) | **<0.001** |  |  |  |  |
| WHO-2022 |  |  |  |  |  | **<0.001** |  | **<0.001** |
| MDS-h, and *SF3B1* |  |  |  |  | Reference | - | Reference | - |
| Low-risk MDS^†^ |  |  |  |  | 0.947 (0.589-1.521) | 0.821 | 0.981 (0.609-1.579) | 0.936 |
| High-risk MDS^‡^ |  |  |  |  | 1.726 (1.013-2.942) | **0.045** | 1.423 (0.828-2.446) | 0.202 |
| MDS-bi*TP53* |  |  |  |  | 4.168 (2.035-8.535) | **<0.001** | 4.729 (2.262-9.886) | **<0.001** |
| IPSS-M |  | **<0.001** |  | **<0.001** |  | **<0.001** |  | **<0.001** |
| Very low/low | Reference | - | Reference |  | Reference | - | Reference | - |
| Moderate low | 1.630 (0.902-2.946) | 0.106 | 1.750 (0.970-3.157) | **0.063** | 1.644 (0.905-2.984) | 0.102 | 1.748 (0.964-3.169) | **0.066** |
| Moderate high | 2.256 (1.301-3.911) | **0.004** | 2.065 (1.183-3.603) | **0.011** | 2.214 (1.259-3.892) | **0.006** | 2.004 (1.130-3.551) | **0.017** |
| High | 2.817 (1.570-5.054) | **0.001** | 2.942 (1.636-5.292) | **<0.001** | 2.931 (1.617-5.313) | **<0.001** | 3.003 (1.650-5.466) | **<0.001** |
| Very high | 5.414 (2.961-9.901) | **<0.001** | 4.847 (2.626-8.944) | **<0.001** | 5.114 (3.336-11.206) | **<0.001** | 5.532 (2.996-10.215) | **<0.001** |
| HMA | 0.885 (0.619-1.264) | 0.501 | 0.761 (0.524-1.105) | 0.152 | 1.007 (0.712-1.425) | 0.968 | 0.890 (0.619-1.279) | 0.528 |
| HSCT | 0.647 (0.369-1.135) | 0.129 | 1.225 (0.734-2.044) | 0.437 | 0.600 (0.344-1.044) | **0.071** | 1.112 (0.673-1.835) | 0.679 |

*P* values of <0.05 are statistically significant.

*As continuous variables analysis.

^†^Low-risk MDS included MDS with del(5q), MDS-*SF3B1*, and MDS, NOS with SLD or MLD.

^‡^MDS/AML with MDS-related gene mutations, MDS-related cytogenetic abnormalities, or not otherwise specified

^§^MDS or MDS/AML with mutated *TP53*

Abbreviations: AMC, absolute monocyte count; CI, confidence interval; EB, excess blasts; HR, Hazard ratios; HMA, hypomethylating agents; HSCT, allogeneic hematopoietic stem cell transplantation; ICC, International Consensus Classification; IPSS-M, Molecular International Prognostic Scoring System; L/M, lymphocyte/monocyte ratio; LFS, leukemia-free survival; MDS, myelodysplastic syndromes/neoplasms; MDS/AML, myelodysplastic syndromes/acute myeloid leukemia; OS, overall survival.

**Supplemental Table 5.** **Multivariable analysis Cox regression analysis of the impact of different variables on the leukemia-free survival and overall survival of patients with myelodysplastic syndromes/neoplasms**

| Variable | LFS | | OS | | LFS | | OS | |
| --- | --- | --- | --- | --- | --- | --- | --- | --- |
|  | **HR (95% CI)** | ***P* value** | **HR (95% CI)** | ***P* value** | **HR (95% CI)** | ***P* value** | **HR (95% CI)** | ***P* value** |
| Age^*^ | 1.025 (1.015-1.036) | **<0.001** | 1.034 (1.022-1.046) | **<0.001** | 1.025 (1.014-1.036) | **<0.001** | 1.033 (1.022-1.045) | **<0.001** |
| Female | 1.233 (0.899-1.692) | 0.194 | 1.144 (0.830-1.576) | 0.410 | 1.249 (0.910-1.715) | 0.169 | 1.157 (0.839-1.595) | 0.374 |
| Ferritin^*^(X 10^2^ ng/mL) | 1.000 (1.000-1.001) | **0.062** | 1.000 (1.000-1.001) | 0.196 | 1.000 (1.000-1.001) | **0.040** | 1.000 (1.000-1.001) | 0.170 |
| AMC | 1.002 (1.000-1.005) | 0.114 | 1.001 (0.998-1.004) | 0.387 | 1.002 (0.999-1.004) | 0.210 | 1.001 (0.998-1.004) | 0.604 |
| ICC |  | **<0.001** |  | **<0.001** |  |  |  |  |
| Low-risk MDS^†^ | Reference | - | Reference | - |  |  |  |  |
| MDS with EB | 1.745 (1.114-2.734) | **0.015** | 1.475 (0.937-2.322) | **0.093** |  |  |  |  |
| MDS/AML^‡^ | 2.603 (1.570-4.317) | **<0.001** | 2.066 (1.231-3.467) | **0.006** |  |  |  |  |
| Mutated *TP53*^§^ | 5.113 (2.701-9.680) | **<0.001** | 6.183 (3.203-11.935) | **<0.001** |  |  |  |  |
| WHO-2022 |  |  |  |  |  | **<0.001** |  | **<0.001** |
| MDS-h, and *SF3B1* |  |  |  |  | Reference | - | Reference | - |
| Low-risk MDS^†^ |  |  |  |  | 0.935 (0.582-1.503) | 0.783 | 0.976 (0.607-1.571) | 0.921 |
| High-risk MDS^‡^ |  |  |  |  | 1.857 (1.101-3.133) | **0.020** | 1.597 (0.944-2.702) | **0.081** |
| MDS-bi*TP53* |  |  |  |  | 4.333 (2.133-8.803) | **<0.001** | 5.035 (2.439-10.392) | **<0.001** |
| IPSS-M |  | **<0.001** |  | **<0.001** |  | **<0.001** |  | **<0.001** |
| Very low/low | Reference | - | Reference |  | Reference | - | Reference | - |
| Moderate low | 1.630 (0.902-2.945) | 0.105 | 1.756 (0.974-3.167) | **0.061** | 1.640 (0.904-2.974) | 0.103 | 1.748 (0.966-3.165) | **0.065** |
| Moderate high | 2.343 (1.354-4.054) | **0.002** | 2.173 (1.248-3.783) | **0.006** | 2.309 (1.318-4.047) | **0.003** | 2.116 (1.199-3.734) | **0.010** |
| High | 2.771 (1.556-4.935) | **0.001** | 2.856 (1.604-5.086) | **<0.001** | 2.880 (1.600-5.184) | **<0.001** | 2.894 (1.605-5.220) | **<0.001** |
| Very high | 5.250 (2.894-9.523) | **<0.001** | 4.597 (2.516-8.399) | **<0.001** | 5.977 (3.289-10.861) | **<0.001** | 5.251 (2.878-9.581) | **<0.001** |
| HMA | 0.882 (0.619-1.259) | 0.490 | 0.756 (0.521-1.097) | 0.141 | 0.997 (0.705-1.412) | 0.988 | 0.868 (0.603-1.249) | 0.445 |
| HSCT | 0.645 (0.368-1.132) | 0.126 | 1.186 (0.710-1.982) | 0.514 | 0.595 (0.342-1.037) | **0.067** | 1.078 (0.651-1.784) | 0.771 |

*P* values of <0.05 are statistically significant.

*As continuous variables analysis.

^†^Low-risk MDS included MDS with del(5q), MDS-*SF3B1*, and MDS, NOS with SLD or MLD.

^‡^MDS/AML with MDS-related gene mutations, MDS-related cytogenetic abnormalities, or not otherwise specified

^§^MDS or MDS/AML with mutated *TP53*

Abbreviations: AMC, absolute monocyte count; CI, confidence interval; EB, excess blasts; HR, Hazard ratios; HMA, hypomethylating agents; HSCT, allogeneic hematopoietic stem cell transplantation; ICC, International Consensus Classification; IPSS-M, Molecular International Prognostic Scoring System; LFS, leukemia-free survival; MDS, myelodysplastic syndromes/neoplasms; MDS/AML, myelodysplastic syndromes/acute myeloid leukemia; OS, overall survival.

**Supplemental Figure 1. Distribution of revised International Prognostic Scoring System (IPSS-R) (A) and molecular IPSS (IPSS-M) (B) for patients with high (>1.5) or low (≤1.5) lymphocyte/monocyte (L/M) ratio**

| (a) |
| --- |
| 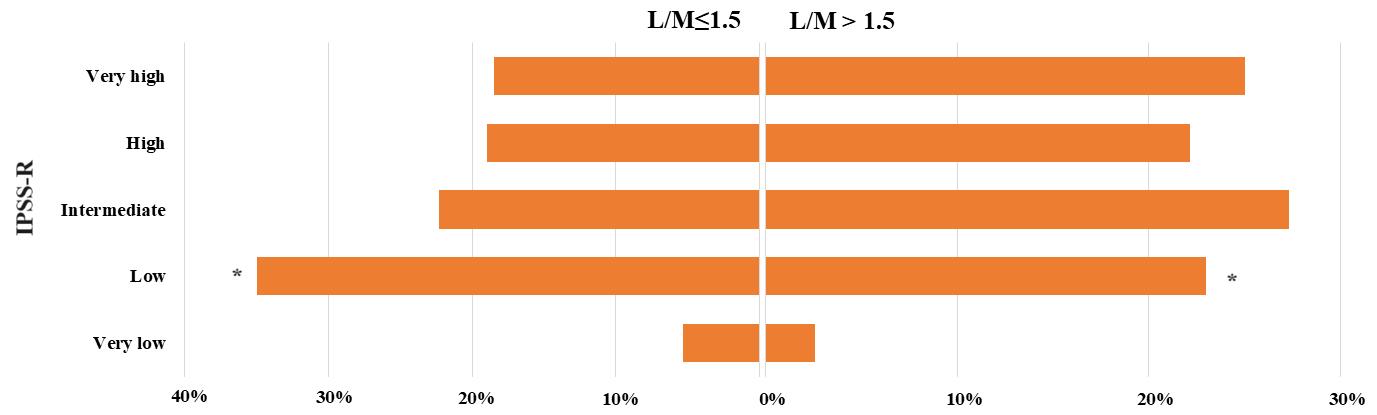 |
| (b) |
| **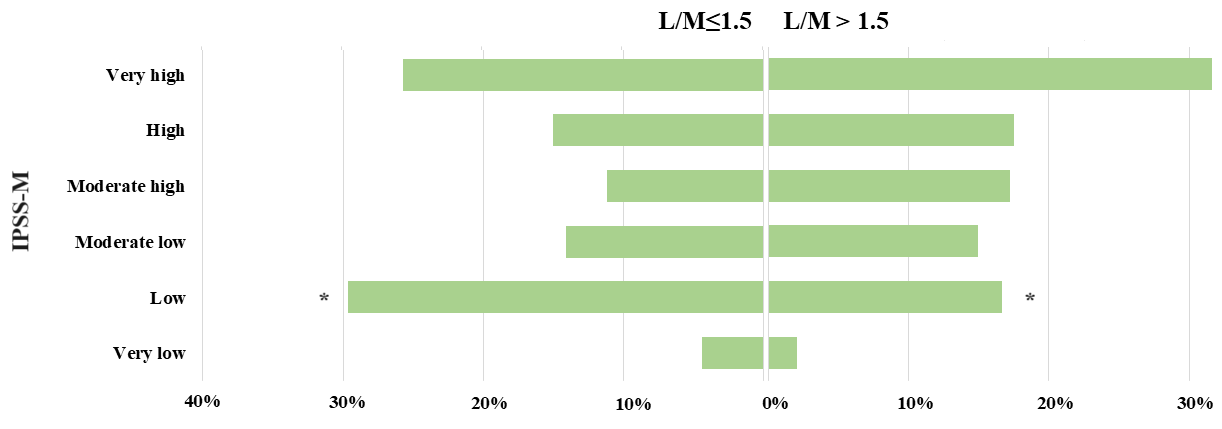** |

Note: *P* value < 0.05 is marked with *

**Supplemental Figure 2. Kaplan-Meier curves for leukemia-free survival and overall survival in patients with myelodysplastic neoplasms/syndromes with or without allogeneic hematopoietic transplantation (HSCT) based on lymphocyte/monocyte (L/M) ratio,**

(A) Leukemia-free survival, stratified by L/M ratio in patients not receiving HSCT

(B) Overall survival, stratified by L/M ratio in patients not receiving HSCT

(C) Leukemia-free survival, stratified by L/M ratio in patients receiving HSCT

(D) Overall survival, stratified by L/M ratio in patients receiving HSCT

| (a) | (b) |
| --- | --- |
| 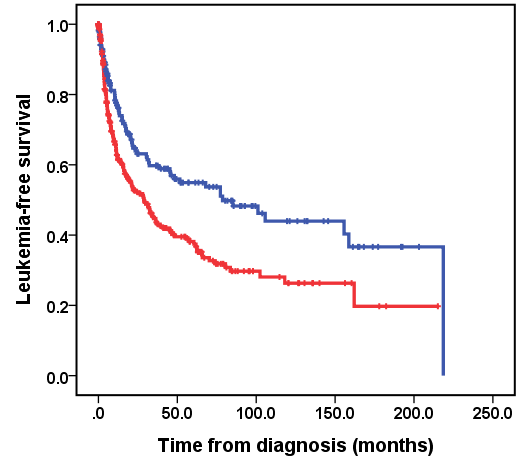  ***P* = 0.001**  **L/M ≤ 1.5, n=175, Median LFS: 78.7 months**  **L/M > 1.5, n=286, Median LFS: 28.8 months** | 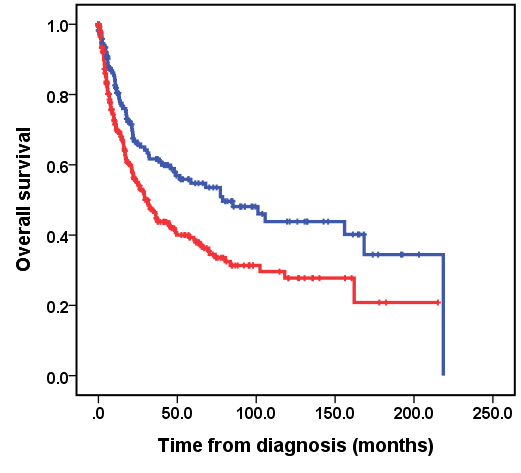  ***P* = 0.001**  **L/M ≤ 1.5, n=175, Median OS: 78.7 months**  **L/M > 1.5, n=286, Median OS: 31.3 months** |
| (c) | (d) |
| 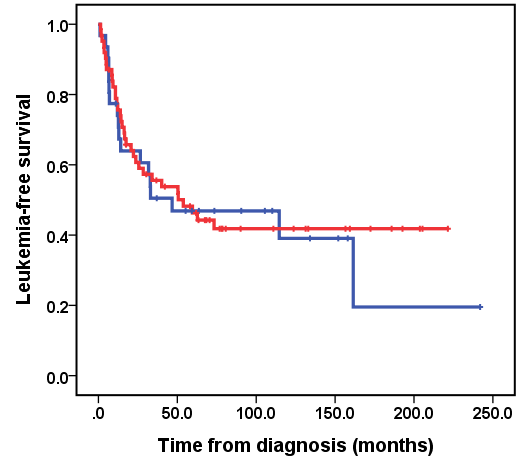  ***P* = 0.755**  **L/M ≤ 1.5, n=31, Median LFS: 46.7 months**  **L/M > 1.5, n=62, Median LFS: 53.8 months** | 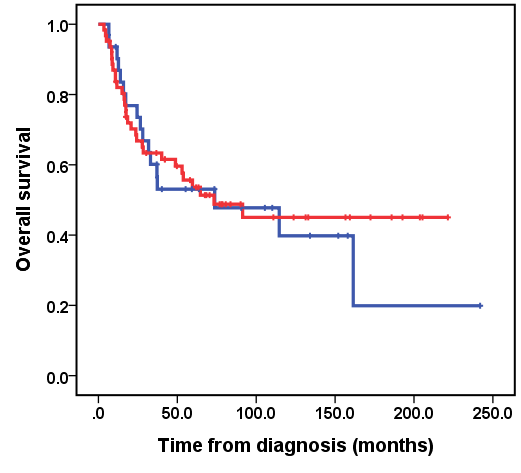  **L/M ≤ 1.5, n=31, Median OS: 73.7 months**  **L/M > 1.5, n=62, Median OS: 73.3 months**  ***P* = 0.759** |

**Supplemental Figure 3. Kaplan-Meier curves for leukemia-free survival and overall survival in patients with very low, low and intermediate-risk revised international prognostic scoring systems with or without allogeneic hematopoietic transplantation (HSCT), stratified by lymphocyte/monocyte (L/M) ratio**

(a) Leukemia-free survival for patients not receiving HSCT

(b) Leukemia-free survival for patients receiving HSCT

(c) Overall survival for patients not receiving HSCT

(d) Overall survival for patients receiving HSCT

| (a) | (b) |
| --- | --- |
| 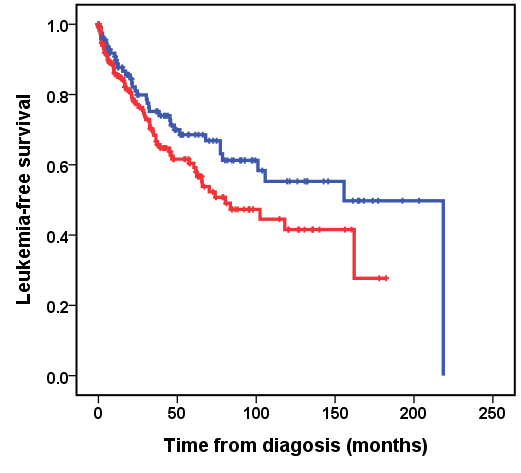  **L/M ≤ 1.5, n=117, Median LFS: 155.7 months**  **L/M > 1.5, n=158, Median LFS: 80.5 months**  ***P* = 0.074** | 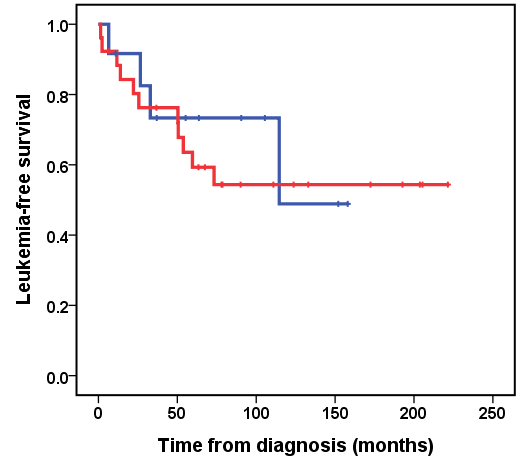  **L/M ≤ 1.5, n=12, Median LFS: 114.6 months**  **L/M > 1.5, n=26, Median LFS not reached**  ***P* = 0.710** |
| (c) | (d) |
| 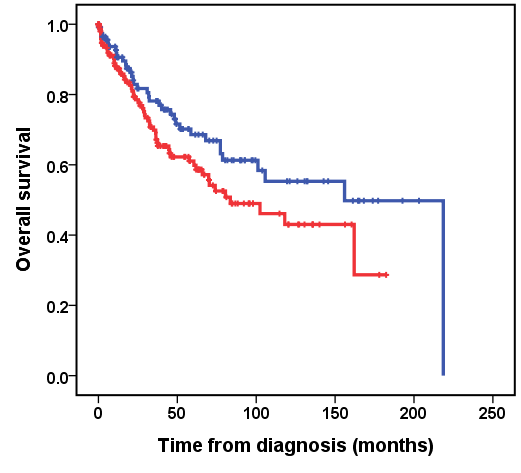  **L/M ≤ 1.5, n=117, Median OS: 156.0 months**  **L/M > 1.5, n=158, Median OS: 83.6 months**  ***P* = 0.079** | 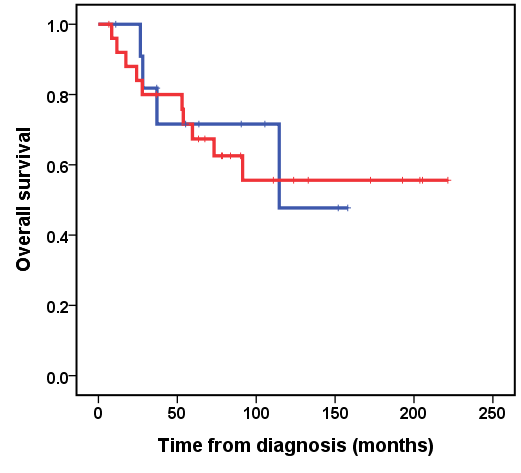  **L/M ≤ 1.5, n=12, Median OS: 114.6 months**  **L/M > 1.5, n=26, Median OS not reached**  ***P* = 0.847** |

**Supplemental Figure 4. Functional analysis of MDS patients with high versus low lymphocyte to monocyte ratio**


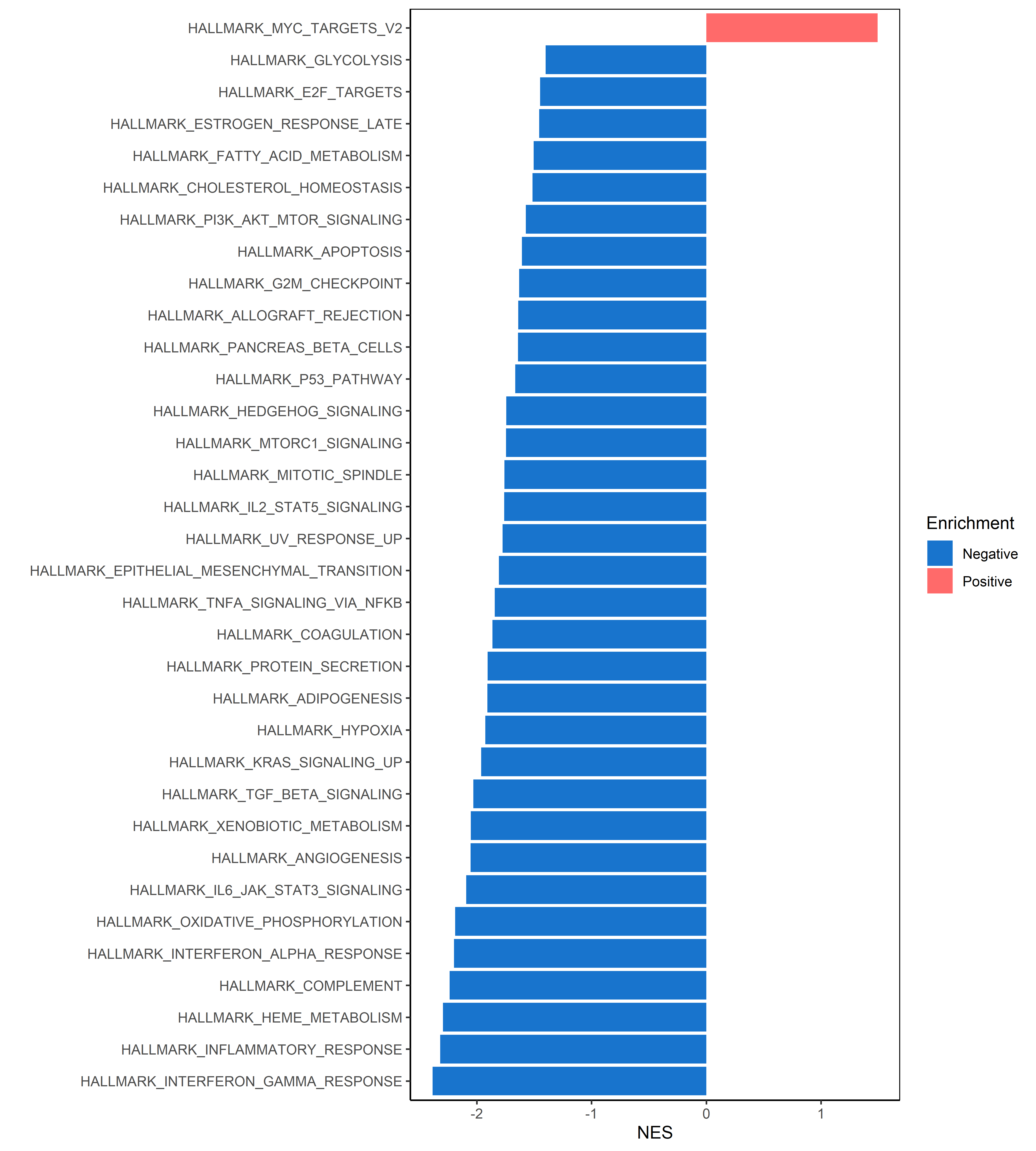

Supplement: Supplementary file 1 — Supplementary Material 1 [file 44313_2025_115_MOESM1_ESM.docx]
